# ^1^H NMR Analysis of Cerebrospinal Fluid from Alzheimer’s Disease Patients: An Example of a Possible Misinterpretation Due to Non-Adjustment of pH

**DOI:** 10.3390/metabo4010115

**Published:** 2014-02-19

**Authors:** Thomas Cruz, Stéphane Balayssac, Véronique Gilard, Robert Martino, Christian Vincent, Jérémie Pariente, Myriam Malet-Martino

**Affiliations:** 1 Université de Toulouse; UPS; Laboratoire de Synthèse et Physico-Chimie de Molécules d’Intérêt Biologique (SPCMIB), Groupe de RMN Biomédicale; 118 route de Narbonne, 31062 Toulouse cedex 9, France; E-Mails: cruz@chimie.ups-tlse.fr (T.C.); balayssac@chimie.ups-tlse.fr (S.B.); gilard@chimie.ups-tlse.fr (V.G.); rmartino@chimie.ups-tlse.fr (R.M.); 2 CHU Purpan; Laboratoire de Biologie cellulaire et Cytologie, Institut Fédératif de Biologie; 330, avenue de Grande-Bretagne; 31059 Toulouse cedex 9, France; E-Mail: vincent.c@chu-toulouse.fr; 3 Université de Toulouse, UPS, CHU Purpan, Imagerie cérébrale et handicaps neurologiques, UMR 825, 31059 Toulouse cedex 9, France; E-Mail: jeremie.pariente@inserm.fr

**Keywords:** ^1^H NMR, cerebrospinal fluid, Alzheimer’s disease, metabolites, chemical shift pH dependence

## Abstract

Two publications from the same research group reporting on the detection of new possible biomarkers for the early diagnosis of Alzheimer’s disease (AD), based on the analysis of cerebrospinal fluid samples (CSF) with ^1^H Nuclear Magnetic Resonance (NMR), are at the origin of the present study. The authors observed significant differences in ^1^H NMR spectra of CSF from AD patients and healthy controls and, thus, proposed some NMR signals (without attribution) as possible biomarkers. However, this work was carried out in non-standardized pH conditions. Our study aims at warning about a possible misinterpretation that can arise from ^1^H NMR analyses of CSF samples if pH adjustment is not done before NMR analysis. Indeed, CSF pH increases rapidly after removal and is subject to changes over conservation time. We first identify the NMR signals described by the authors as biomarkers. We then focus on the chemical shift variations of their NMR signals as a function of pH in both standard solutions and CSF samples. Finally, a principal component analysis of ^1^H NMR data demonstrates that the same CSF samples recorded at pH 8.1 and 10.0 are statistically differentiated.

## 1. Introduction

Alzheimer’s disease (AD) is one of the leading causes of progressive dementia in the elderly. To date, diagnosis and/or prognosis of AD remain difficult and rely on clinical criteria for dementia. The search for biofluid metabolic biomarker(s) of which concentration is likely to change at an early stage of the disease is, thus, of great interest, especially in the cerebrospinal fluid (CSF) as it is a material routinely obtained for diagnosing AD whose composition reflects metabolite production in the brain. To this aim, proton Nuclear Magnetic Resonance (^1^H NMR) has many assets, despite its low intrinsic sensitivity. Indeed, it is a high-throughput, quantitative, robust, and highly reproducible method that provides a comprehensive measurement of the low molecular weight components of the biofluid analyzed. The wealth of chemical information provided by NMR arises from the dependence of the chemical shifts of NMR-active nuclei on their chemical environment. However, the positions of the NMR resonances are sensitive to subtle differences in some physicochemical parameters such as pH, temperature, ionic strength, *etc*. In particular, the protonation state of an acidic or basic site has a strong influence on the chemical shifts of protons close to ionizable group(s). A careful control of pH is, thus, required when metabolic profiles of samples have to be compared.

In two recent articles, Kork *et al.* [1,2] proposed potential new biomarkers for the early diagnosis of AD. Their study was based on the analysis of CSF from AD patients and healthy control subjects by the means of ^1^H NMR. They demonstrated that ^1^H NMR spectra of CSF from AD patients showed specific signals that could not be detected in the majority of CSF from healthy subjects, or signals of variable intensity between AD patients and healthy controls. They concluded that “^1^H NMR is obviously a capable method for detection and quantification of substances in the CSF of AD patients even without the knowledge of molecular structures” [1]. In the present study, we show that the lack of pH standardization of the samples analyzed is responsible for most of the differences observed by the authors in the ^1^H NMR spectra of CSF from healthy subjects and AD patients.

## 2. Results and Discussion

### 2.1. Assignment of ^1^H NMR Signals

**Figure 1:**
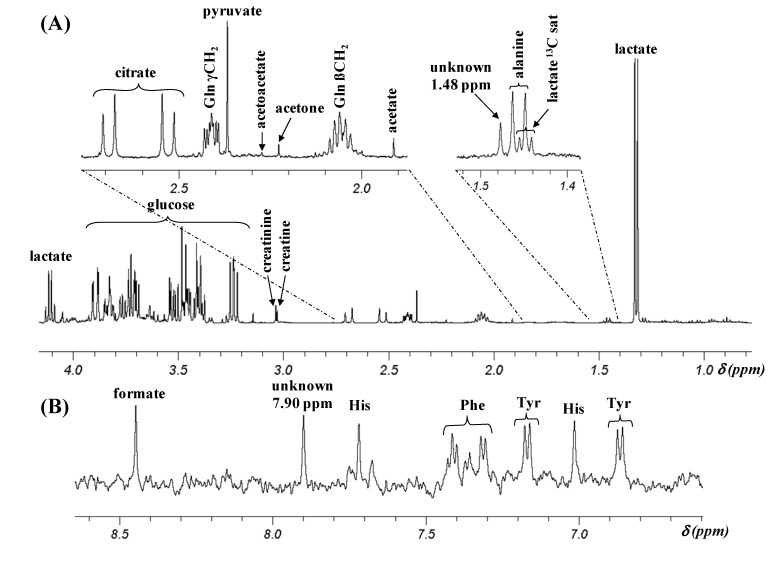
Representative ^1^H NMR spectrum of a CSF sample containing 33% D_2_O recorded at 298K and pH 8.7. (**A**) 0.8–4.2 ppm region, LB = 0.3 Hz; (**B**) 6.6–8.6 ppm region, LB = 2.5 Hz. Gln: glutamine; sat: satellite; His: histidine; Phe: phenylalanine; Tyr: tyrosine.

Figure 1 illustrates a representative ^1^H NMR spectrum of a CSF sample at pH 8.7 from an AD patient, with particular emphasis on signals with chemical shifts (δ) between 1.4 and 2.8 ppm and between 6.6 and 8.6 ppm, the two regions of interest in the studies of Kork *et al.* [1,2].

**Figure 2:**
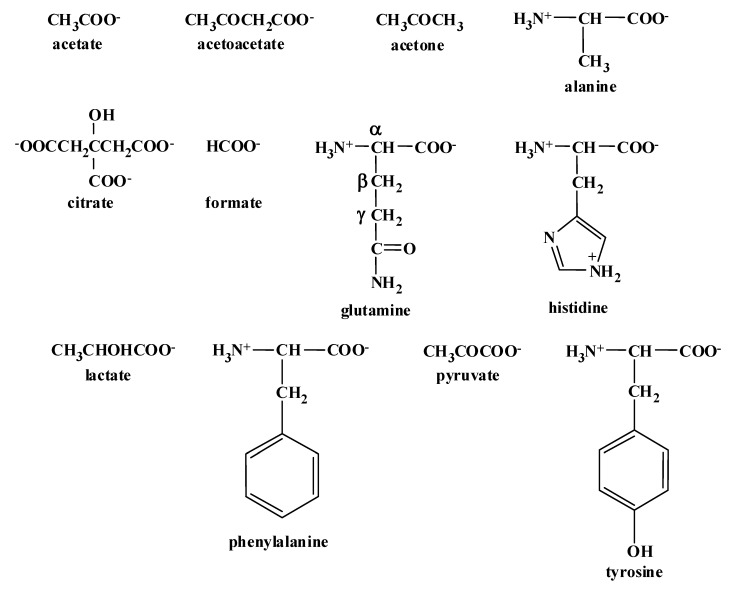
Chemical structures of the CSF metabolites discussed in this study.

The aliphatic region displays the resonances of citrate (AB system, δ_A_ 2.69 ppm and δ_B_ 2.53 ppm, *J*_AB_ = 16.2 Hz), glutamine (two multiplets (m) centered at 2.41 and 2.06 ppm), pyruvate (singlet (s) at 2.37 ppm), acetotacetate (s at 2.27 ppm), acetone (s at 2.23 ppm), acetate (s at 1.91 ppm), an unknown metabolite (s at 1.48 ppm), alanine (doublet (d) at 1.46 ppm, *J* = 7.2 Hz) and lactate (d at 1.32 ppm, *J* = 6.9 Hz) with its downfield ^13^C satellite peaks at 1.46 and 1.43_5_ ppm (d, J = 6.9 Hz). The aromatic part of the spectrum includes the signals of three amino-acids: histidine (two s at 7.71 and 7.01 ppm), phenylalanine (three multiplets centered at 7.41, 7.35 and 7.32 ppm), and tyrosine (AA’XX’ system with two apparent doublets at 7.16 and 6.86 ppm, *J*_AX_ = *J*_A’X’_ = 8.2 Hz) as well as those of formate (s at 8.45 ppm) and of an unknown metabolite (s at 7.90 ppm). The assignments of these resonances are in agreement with literature data ([3,4], and the Human Metabolome Database Version 3.5 at http://www.hmdb.ca/ [5]). The chemical structures of these metabolites are illustrated in Figure 2.

### 2.2. pH Dependence of ^1^H Chemical Shifts

Except acetone, all the compounds cited above have ionizable groups (principally carboxylic acids and α-amino acids, with pK_a_ values between 1.8 and 9.7) of which ionization state affects the chemical shifts. Unlike plasma, CSF has a poor buffering capacity and rapid increase in pH occurs once it is removed from an atmosphere with a physiological level of CO_2_, from 7.4–7.6 at the time of sampling to 7.8–9.1 within 60 s and ~9.6 after 2 h at 4 °C [6]. A pH rise to 9.2–9.6 was observed in samples stored at −20 °C immediately after sampling. Indeed, with CO_2_ (solid), having the ability to sublimate to CO_2_ (gas) at temperatures above −78 °C, its evaporation induces pH increase [7]. For samples stored at −80 °C, Strawn *et al.* [8] reported that there was no pH change after 30 days and no significant correlation between pH and storage time in the range 72 h–118.3 months. Nevertheless, they observed a pH increase at 4 °C (from 8.2 to 8.5 over 12 h), as well as during repeated freeze-thaw cycles.

**Figure 3:**
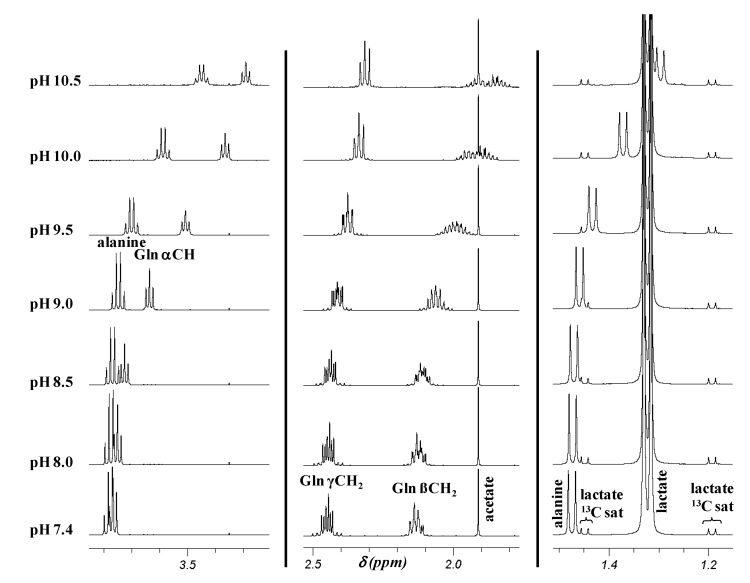
Partial ^1^H NMR spectra (1.15–3.9 ppm region) of a model solution containing acetate, alanine, glutamine and lactate in D_2_O at various pHs between 7.4 and 10.5. Gln: glutamine; sat: satellite.

pHs of the 23 CSF samples analyzed in this study and measured just before recording of their NMR spectra are in accordance with these findings: their values range from 8.0 to 9.6 (mean 8.9 ± 0.5). In this pH range, the ^1^H δ of glutamine, alanine, phenylalanine, tyrosine, and histidine with pK_a_ values of the protonated amino group between 9.1 and 9.7 are sensitive to the pH of the samples. In contrast, those of the other metabolites do not vary because their carboxylic acid group is under carboxylate form. To determine the δ variation of the five amino acids and to demonstrate the insensitivity to pH of carboxylic acid δ, the spectra of two model solutions (the first containing acetate, alanine, glutamine and lactate and the second, formate, histidine, phenylalanine, and tyrosine), were recorded at physiological pH and from pH 8.0 to 10.5 by step of 0.5 units. The spectra of the first model solution are displayed in Figure 3.

As expected, the δ of the signals of formate (s at 8.45 ppm), lactate (quadruplet at 4.10 ppm, *J* = 6.9 Hz and d at 1.32 ppm, *J* = 6.9 Hz), and acetate (s at 1.91 ppm) do not change over the pH range while those of the amino acids are shifted upfield as a consequence of deprotonation of the ammonium group with increasing pH, especially since the protons are near the site of protonation. Indeed, the α CH triplet of glutamine is shielded by 0.48 ppm, from 3.77 ppm at physiological pH to 3.29 ppm at pH 10.5, while in the same pH range, the β and γ CH_2_ multiplets are shielded respectively by 0.25 ppm (from 2.13 to 1.88 ppm) and 0.14 ppm (from 2.45 to 2.31 ppm) (Table 1). Moreover, the shape of the CH_2_ resonances changes as a function of pH. The β CH_2_ multiplet turns out more split with pH increase whereas the γ CH_2_ multiplet becomes a “triplet” at pH 10 and 10.5. The CH quadruplet and CH_3_ doublet of alanine are shielded respectively by 0.33 and 0.17 ppm, from 3.78 and 1.47 ppm at pH 7.4 to 3.45 and 1.30 ppm at pH 10.5 (Table 1).

The aromatic protons of histidine, phenylalanine, and tyrosine, which are separated from the NH_3_^+^/NH_2_ moiety by at least five bonds, are shielded between pH 7.4 and 10.5 by only ~0.1 ppm for histidine, 0.05 ppm for phenylalanine, and 0.13–0.19 ppm for tyrosine, as, at pH > 9.5, the contribution due to the deprotonation of the phenol group (pK_a_ = 10.1) must be added (Table 1).

**Table 1:** Chemical shifts (δ) of some cerebrospinal fluid samples (CSF) metabolites discussed in this study as a function of pHs in the range 7.4–10.5.

Metabolite^c^	pH 7.4 ^a^		pH 9.5 ^a^		pH 10.5 ^a^		Upfield shift ^b^	
	In model solution	In CSF	In model solution	In CSF	In model solution	In CSF	In model solution	In CSF
Acetate								
CH_3_ s	1.91	1.91	1.91	1.91	1.91	1.91	0	0
Alanine								
CH_3_ d	1.47	1.47	1.43	1.42	1.30	1.29	0.17	0.18
CH q	3.78	ND ^d^	3.70	ND ^d^	3.45	ND ^d^	0.33	ND ^d^
Formate								
CH s	8.45	8.45	8.45	8.45	8.45	8.45	0	0
Glutamine								
α CH t	3.77	ND^d^	3.51	ND ^d^	3.29	ND ^d^	0.48	/
β CH_2_ m	2.13	2.13	1.99	1.98	1.88	1.87	0.25	0.26
γ CH_2_ m	2.45	2.45	2.37	2.36	2.31	2.31	0.14	0.14
Histidine								
Ar CH s	7.05	7.05	6.98	6.99	6.92	6.92	0.13	0.13
Ar CH s	7.77	7.77	7.69	7.69	7.67	7.67	0.10	0.10
Lactate								
CH_3_ d	1.32	1.32	1.32	1.32	1.32	1.32	0	0
CH q	4.10	4.11	4.10	4.11	4.10	4.11	0	0
Phenylalanine								
ortho CH m	7.33	7.33	7.31	7.30	7.29	7.29	0.04	0.04
para CH m	7.37	7.37	7.34	7.33	7.31	7.31	0.06	0.06
meta CH m	7.42	7.42	7.40	7.40	7.38	7.38	0.04	0.04
Tyrosine								
Ar CH app d	6.89	6.89	6.83	6.83	6.70	6.69	0.19	0.20
Ar CH app d	7.19	7.19	7.14	7.15	7.06	7.05	0.13	0.14

^a^ ^1^H NMR spectra are recorded in D_2_O for model solutions, and for CSF after addition of 150 μL of D_2_O to 300 mL of CSF. The pH indicated is the pH-meter reading value. Although the chemical shifts are measured at seven different pHs, only those at the two extreme pHs (7.4 and 10.5) and at pH 9.5 (chosen as intermediate between the pKa_(NH3_^+^_/NH2)_ values of glutamine, histidine, phenylalanine and tyrosine (~9.1) and alanine (9.7)) are reported; ^b^ Upfield shift between pHs 7.4 and 10.5; ^c^ Chemical shifts are expressed in ppm. Ar: aromatic; d: doublet; m: multiplet; q: quadruplet; s: singlet; t: triplet; app: apparent; ^d^ ND: not determined because of the overlap of resonances with those of other CSF metabolites.

**Figure 4:**
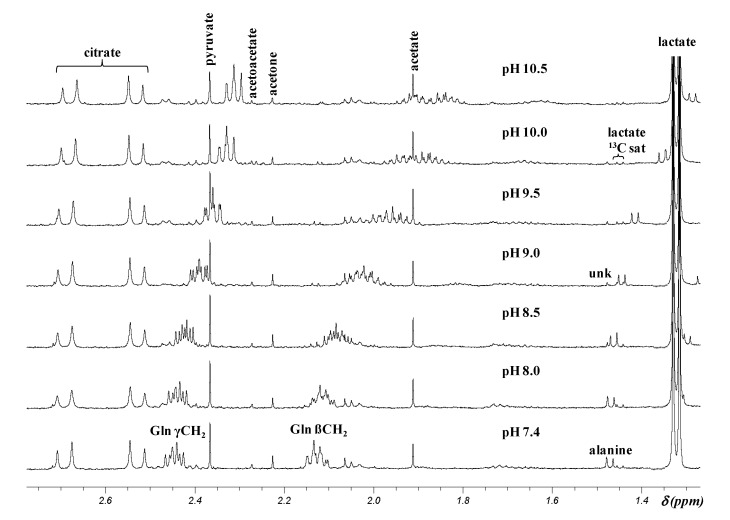
Representative ^1^H NMR spectra (1.25–2.8 ppm region) of a CSF sample containing 33% D_2_O whose pH was adjusted at different pH between 7.4 and 10.5. Gln: glutamine; sat: satellite; unk: unknown.

The δ of lactate, β and γ CH_2_ multiplets of glutamine, acetate, CH_3_ doublet of alanine, formate, and aromatic resonances of histidine, phenylalanine, and tyrosine are almost identical (within 0.01 ppm) in an AD patient CSF sample in which pH was adjusted to 7.4 and then from 8 to 10.5 by step of 0.5, to those determined in the model solutions (Table 1 and Figure 4 for the 1.25–2.8 ppm region of the ^1^H NMR spectrum).

### 2.3. Principal Component Analysis (PCA) of the ^1^H NMR Data from CSF Samples at Two Different pH

**Figure 5:**
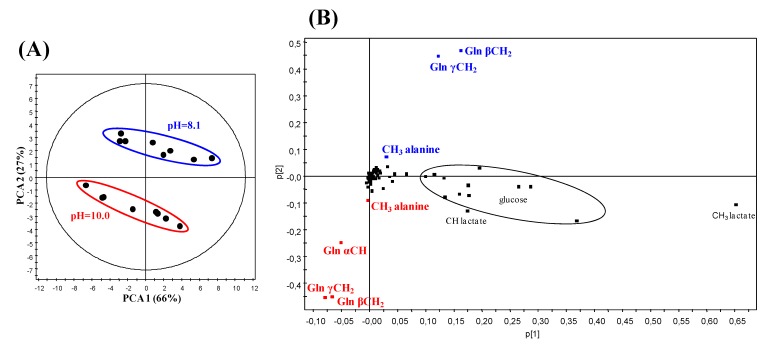
Score plot (**A**) and loading plot (**B**) of a Principal Component Analysis (PCA) applied to ^1^H NMR spectra of eight CSF samples from AD patients recorded successively at pH 8.1 ± 0.1 and 10.0 ± 0.1. The principal discriminating metabolites are written in blue for pH 8.1 and red for pH 10.0.

pH increase during CSF collection, handling and storage combined to the strong pH dependence of the chemical shits of numerous CSF metabolites requires a careful adjustment of pH values before ^1^H NMR spectra recording, e.g., at 2.5, 7.0, or 9.5 [3,9,10], to improve recognition and assignment of resonances. The absolute necessity to standardize the pH of CSF samples in order to determine whether ^1^H NMR spectral differences exist between healthy and diseased groups is illustrated with the following experiment. Principal Component Analysis (PCA), a classical unsupervised multivariate statistical method, was applied to the ^1^H NMR data of eight CSF from AD patients recorded after pH adjustment at two different values, 8.1 ± 0.1 and 10.0 ± 0.1. The resulting PCA score plot shows two clearly separated clusters with the principal components 1 and 2, explaining 93% of the variation in the areas of metabolite signals (Figure 5A). The loading plot highlights that the variables contributing to this separation are those corresponding to resonances of glutamine and alanine to a lesser extent. Indeed, the signals of α CH and β and γ CH_2_ of glutamine, as well as the CH_3_ doublet of alanine at pH 10.0, come out in the lower part of the plot whereas the β and γ CH_2_ multiplets of glutamine and the CH_3_ doublet of alanine at pH 8.1 appear in the upper part (Figure 5B); the α CH glutamine triplet overlapping with high intensity glucose resonances at this pH is not observed. This analysis shows that the same samples can be discriminated depending on their pH. It is, therefore, mandatory to standardize the pH of CSF samples before ^1^H NMR measurements in order to avoid that raw data processing and their subsequent statistical analysis lead to erroneous conclusions.

### 2.4. Effect of pH on the δ of the Resonances Proposed as Biomarkers by Kork et al.

Among the characteristic resonances discussed by Kork *et al.* [1,2], only those at 1.92 and 8.46 ppm that can be attributed to acetate and formate, respectively, are insensitive to pH of CSF samples. Acetate and formate signals are respectively observed at 1.91 and 8.45 ppm in our study. Thus, the δ reported by Kork *et al.* can be directly compared to those we determined in the model solutions and in the CSF samples as a function of pH. 

The two multiplets at 2.15 and 2.45 ppm proposed by the authors as early biomarkers of AD since they occurred in 19 out of 20 CSF samples of AD patients, whereas, they were observed in only 11 (for the signal at 2.15 ppm) and eight (for the resonance at 2.45 ppm) out of 27 CSF samples from healthy subjects, can be identified respectively as the β and γ CH_2_ resonances of glutamine in a CSF sample at pH ~8.0 (Table 1, Figure 4). Their absence in the majority of CSF samples from healthy control subjects results from pH changes. Indeed, the comparison of the spectrum of a healthy subject CSF sample reported by Kork *et al.* in their Figure 1B [1] to the spectra of standard glutamine in D_2_O or of a CSF sample whose pHs were adjusted at different values between 7.4 and 10.5 (Figure 3 and Figure 4), shows that glutamine β and γ CH_2_ resonances are present in their Figure 1B [1] at ~2.05 and 2.4 ppm. From the δ and multiplicity of these signals, it can be concluded that the pH of the CSF sample analyzed was between 9.0 and 9.5. Thus, the presence or absence of the multiplets at 2.15 and 2.45 ppm in non-standardized pH CSF samples is not a biomarker of AD as claimed by the authors [1] because the chemical shifts of these resonances that belong to glutamine are pH-dependent.

In their second study, Kork *et al.* [2] were interested in the two resonances at 1.44 and 1.47 ppm. This spectrum region displays two peaks corresponding to the methyl doublet of alanine with pH-dependent δ in the pH range of CSF, as well as two or three much less intense peaks corresponding to the downfield ^13^C satellite doublet of the methyl doublet of lactate at 1.44 and 1.45_5_ ppm, and to an unassigned singlet, not always detected, at 1.48 ppm (Figure 1). Both, the satellite doublet and the singlet, are insensitive to pH variation of CSF samples. The singlet signal at 1.48 ppm and the downfield peak of alanine methyl doublet overlap at pH ~ 7.4–8.0 (Figure 4), whereas the four signals of the methyl doublets of alanine and downfield lactate ^13^C satellite can be partially or totally superimposed at pHs between ~8.5 and ~9.5 (Figure 3 and Figure 4).

To which metabolite(s) do the resonances at 1.44 and 1.47 ppm correspond? Their chemical shift difference (0.03 ppm) precludes their attribution to the two peaks of the methyl doublet of alanine (*J* = 7.2 Hz) or lactate ^13^C satellite (*J* = 6.9 Hz), which should be distant of 0.014 ppm with a 500 MHz spectrometer. Moreover, from the chemical shift values and/or shape of glutamine resonances, it can be estimated that 19 over 20 AD CSF samples analyzed by Kork *et al.* [1] had a pH ~ 8, whereas the pH of most of the healthy subject samples was more basic, up to >9. Thus, it can be deduced, from the spectra of the first model solution and CSF sample at various pHs, that the two peaks of the alanine doublet would appear at 1.47_5_ for the first one and 1.46 ppm for the second one in all but one CSF samples of AD patients, and at around 1.45 and 1.43_5_ ppm (overlapping with the peaks of the lactate satellite doublet at 1.45_5_ and 1.44 ppm) in most of healthy subject CSF samples (Figure 3 and Figure 4). Hence the signals at 1.47 and 1.44 ppm correspond to the methyl signals of alanine at pH ~ 8 and pH ~ 9 respectively, whatever the resonance of each doublet considered by the authors. This attribution is confirmed by the fact that the intensity of the signal at 1.47 ppm was superior to that of the signal at 1.44 ppm in CSF samples of AD patients and lower in those of healthy subjects as reported by Kork *et al.* [2] in their Table 2. Therefore, on no account, these resonances that are both those of alanine at different pHs can be biomarkers of AD.

### 2.5. Quantification of Some CSF Metabolites Discussed in this Study

CSF glutamine and alanine concentrations determined in our study showed no significant differences between AD patients and controls (Table 2), in agreement with the LC-MS/MS studies conducted by Fonteh *et al.* [11] (only for glutamine as alanine was not assayed) and Czech *et al.* [12], but not with the results of Mochizuki *et al.* [13] who found a significantly higher concentration of alanine and D’Aniello *et al.* [14] who reported a significantly higher level of glutamine but not of alanine in AD CSF compared to normal CSF.

As stated above, the resonance at 1.92 ppm that corresponds to acetate is insensitive to pH differences of CSF samples. It was considered as a possible biomarker of AD by Kork *et al.* [2] who found that the intensity of its signal dropped in AD patients. A significantly lower CSF concentration of acetate (p = 2 × 10^−4^) was also reported by Blasco *et al.* [9] in amyotrophic lateral sclerosis patients compared to controls. As Nicoli *et al.* [15], we observe no significant difference in acetate concentration between AD patients and control subjects (Table 2).

Kork *et al.* (Table 2 in [2]) listed several resonances in the chemical shift range 6.6–8.6 ppm. From the results of our study, they can be attributed to tyrosine (two apparent doublets with signals at 6.87 and 6.89 ppm on one hand and at probably 7.17 (not reported in the Table but clearly visible in the spectrum of Figure 1A [2]) and 7.19 ppm on the other hand), histidine (two singlets at 7.03 and 7.73 ppm), phenylalanine (complex resonance pattern with signals measured at 7.33, 7.43, and 7.44 ppm), formate (singlet at 8.46 ppm), and an unknown compound (singlet at 7.91 ppm). The resonances of the three amino acids and of the unknown metabolite had a significantly higher intensity in AD patients compared to healthy subjects and were considered by the authors as biomarkers of AD [1,2]. It is very surprising that they have not been detected in most of the healthy subjects (20 or 21 over 27). Indeed, Stoop *et al.* [16] reported mean concentrations determined by ^1^H NMR and LC-MS/MS of 9.5 and 11.2 μM for tyrosine, 11.5 and 17.2 μM for histidine, and 8.8 and 9.0 μM for phenylalanine in normal human CSF, which agree well with the values determined by Wishart’s group [5,17,18]. Our results reported in Table 2 are in accordance with these last data. Moreover, the mean concentrations we determined for these three metabolites are not significantly different in AD patients and control subjects: respectively 11.0 and 11.9 μM for tyrosine, 13.5 and 13.4 μM for histidine, and 15.3 and 15.1 μM for phenylalanine (Table 2), in agreement with most of the literature reports. Indeed, no significant changes between control and AD patients were observed for these three amino acids [11,14,15,19], except for tyrosine of which the level significantly increased in demented patients [15] and for tyrosine and phenylalanine that were found significantly different in female but not in male AD patients [12]. Kork *et al.* [2] reported a significant decreased intensity of the formate resonance in AD patients. This result although not in line with a previous study that revealed a significant higher level of formate in demented patients compared to control patients [15] is consistent with our own data (Table 2).

**Table 2:** Concentrations of selected CSF metabolites in Alzheimer’s disease (AD) patients (n = 15) and control subjects (n = 8).

Metabolite	AD patients	Control	*p*-value ^a^	Literature value in normal CSF (μM) [18]
	Mean ± sd (μM)	Mean ± sd (μM)		
Acetate	57 ± 23	47 ± 32	0.21	58 ± 27 ^b^
Alanine	48 ± 18	46 ± 17	0.55	46 ± 27 ^b^
Formate	28 ± 10	43 ± 15	0.01	32 ± 16 ^b^
Glutamine	532 ± 67	543 ± 137	0.51	398 ± 150 ^b,c^
Histidine	14 ± 3	13 ± 3	0.73	15 ± 8 ^b,c^
Phenylalanine	15 ± 4	15 ± 5	0.73	15 ± 8 ^b,c^
Tyrosine	11 ± 3	12 ± 5	0.73	12 ± 9 ^b^

^a^ Wilcoxon test; ^b^ Metabolite concentration measured by ^1^H NMR; ^c^ Metabolite concentration measured by direct flow injection-MS/MS.

## 3. Experimental Section

### 3.1. Patients and CSF Sampling

This study was conducted on 23 patients recruited from University Hospital Purpan in Toulouse, France. All the patients signed a form of information and absence of opposition to the use of their biological samples for research purposes, a procedure approved by the Comité de Protection des Personnes “Sud-Ouest et Outre-mer”. All the patients had a lumbar puncture for diagnosis. Fifteen presented an AD (AD group) and 8 did not suffer from any inflammatory, degenerative, or tumoral disease of the central nervous system (control group). After the lumbar puncture, the CSF samples were put on ice for transportation, centrifuged (3000 rpm for 10 min at 4 °C) and frozen at −80 °C until ^1^H NMR analysis.

### 3.2. Chemicals

Sodium acetate, lithium acetoacetate, acetone, alanine, citric acid monohydrate, ammonium formate, glutamine, sodium lactate, sodium pyruvate, and the NMR reference 2,2,3,3-d_4_-3-(trimethylsilyl)propionic acid sodium salt (TSP) were purchased from Sigma-Aldrich (France). l-histidine, L-phenylalanine and L-tyrosine were obtained from Acros Organics (France). D_2_O, NaOD and DCl solutions used for pH adjustments were supplied by Eurisotop (France).

### 3.3. Preparation of CSF Samples and Model Solutions

#### 3.3.1. CSF Samples

A volume of 150 μL of D_2_O was added to 300 μL of CSF. After measuring pH, the solution was transferred into a 5 mm diameter NMR ultra-glass tube. A sealed coaxial capillary containing a solution of TSP, which served as external chemical shift and quantification reference, was placed in the NMR tube. This protocol was used for the NMR recording of CSF samples at natural pH. For experiments in which the pH of the CSF sample was modified, the same protocol was followed except that pH was adjusted to the desired value by adding 1 to 5 μL of a diluted solution of NaOD or DCl before recording ^1^H NMR spectra. All pH values refer to pH-meter reading uncorrected for the deuterium isotope effect.

#### 3.3.2. Model Solutions

Two model solutions were prepared in D_2_O. The first one contained acetate sodium salt (1.6 mM), alanine (6.4 mM), glutamine (5.0 mM), and lactate sodium salt (99.5 mM), and the second, formate ammonium salt (16.5 mM), histidine (5.2 mM), phenylalanine (5.0 mM), and tyrosine (5.0 mM). Each solution was divided into seven aliquots whose pHs (uncorrected pH-meter reading values) were adjusted to 7.4, 8.0, 8.5, 9.0, 9.5, 10.0, and 10.5 (±0.1) by addition of a diluted NaOD solution.

### 3.4. ^1^H NMR Spectra Recording Conditions

^1^H NMR experiments were performed at 298K on a Bruker Avance 500 spectrometer operating at a proton frequency of 500.13 MHz and equipped with a 5 mm cryoprobe. One dimensional pulse-acquire spectra were recorded using a pulse width of 3.4 μs (flip angle: 30°) with a 2.0 s water presaturation delay. FIDs were acquired over a sweep width of 16 ppm (8,000 Hz) into 64K data points during an acquisition time of 4.09 s, resulting in a repetition time of 6.09 s, and 256 scans were collected. Prior to Fourier transformation, the FIDs were multiplied by an exponential line-broadening function of 0.7 Hz (unless otherwise indicated). Under these recording conditions, the ^1^H resonances were considered as fully relaxed since no significant change in the signal intensities was observed with a repetition time of 11.09 s. All spectra were referenced by setting the ^1^H δ of the TSP methyl groups to zero.

Metabolite concentrations were measured by comparing the expanded areas of their respective NMR signals with that of the TSP methyl protons singlet. The apparent concentration of the TSP reference was previously determined against solutions of succinate and maleate of precisely known concentrations in the same recording conditions as those described above. The significance of the differences in metabolite concentrations between AD and control groups was assessed with the Wilcoxon test.

Signal assignment was achieved with an in-house database established by considering the effects of pH variations on chemical shits and multiplicity of ^1^H NMR resonances, and confirmed by spiking representative CSF samples with authentic standards.

### 3.5. Statistical Analysis

^1^H NMR spectra were transferred to the KnowItAll® software (Bio-Rad) where baseline correction was performed on each spectrum. The bin area method was used to segment the spectra between −0.2 and 8.8 ppm using the intelligent variable size bucketing tool included in the KnowItAll® package. The bins of H_2_O/HOD (4.2–6.2 ppm) resonance were excluded. A manual filtering procedure was applied to the whole spectrum to exclude buckets that contained only noise. Buckets corresponding to one metabolite were then grouped when possible, according to NMR signal assignment. A total of 74 variables were, thus, retained for the subsequent statistical analysis. Bin areas were integrated and transferred to the Pirouette software (Infometrics) and a matrix consisting of rows that identify samples and columns that represent variables (metabolites) was generated. Integrated regions were normalized by dividing their areas by that of the external standard TSP. Data were preprocessed by mean-centering and the new matrix thus obtained was subjected to PCA analysis with the SIMCA-P+ 12.0 software (Umetrics, Umeå, Sweden).

## 4. Conclusions

As CSF is poorly buffered, its pH increases rapidly after removal from the body and is also subject to changes during storage at temperatures above −78 °C. The “natural” pH of CSF samples is thus variable (between 8.0 and 9.6 for the 23 samples analyzed in this study). Moreover, the ^1^H NMR chemical shifts of the metabolites with ionizable groups in the pKa range ~7–10 depend on the pH. Thus, recording ^1^H NMR spectra of CSF samples at their “natural” pH without adjustment at a given value can lead to incorrect assignment of the resonances and to wrong conclusions. Furthermore, the identification of the metabolite(s) that give(s) rise to the ^1^H NMR signals proposed as potential biomarkers is necessary to confirm their relevance.

## References

[1] Kork F., Holthues J., Hellweg R., Jankowski V., Tepel M., Öhring R., Heuser I., Bierbrauer J., Peters O., Schlattmann P. (2009). A possible new diagnostic biomarker in early diagnosis of Alzheimer's disease. Curr. Alzheimer Res..

[2] Kork F., Gentsch A., Holthues J., Hellweg R., Jankowski V., Tepel M., Zidek W., Jankowski J. (2012). A biomarker for severity of Alzheimer’s disease: ^1^H-NMR resonances in cerebrospinal fluid correlate with performance in mini-mental-state-exam. Biomarkers.

[3] Wevers R.A., Engelke U., Wendel U., de Jong J.G.N., Gabreëls F.J.M., Heerschap A. (1995). Standardized method for high-resolution ^1^H-NMR of cerebrospinal fluid. Clin. Chem..

[4] Lutz N.W., Viola A., Malikova I., Confort-Gouny S., Ranjeva J.P., Pelletier J., Cozzone P.J. (2007). A branched-chain organic acid linked to multiple sclerosis: First identification by NMR spectroscopy of CSF. Biochem. Biophys. Res. Comm..

[5] Wishart D.S., Jewison T., Guo A.C., Wilson M., Knox C., Liu Y., Djoumbou Y., Mandal R., Aziat F., Dong E. (2013). Hmdb 3.0-The human metabolome database in 2013. Nucleic Acids Res..

[6] Cunniffe J.G., Whitby-Strevens S., Wilcox M.H. (1996). Effect of pH changes in cerebrospinal fluid specimens on bacterial survival and antigen test results. J. Clin. Pathol..

[7] Wuolikainen A., Hedenström M., Moritz T., Marklund S.L., Antti H., Andersen P.M. (2009). Optimization of procedures for collecting and storing of CSF for studying the metabolome in ALS. Amyotroph. Lateral Scler..

[8] Strawn J.R., Ekhator N.N., Geracioti T.D. (2001). In-use stability of monoamine metabolites in human cerebrospinal fluid. J. Chromatogr. B Biomed. Sci. Appl..

[9] Blasco H., Corcia P., Moreau C., Veau S., Fournier C., Vourc’h P., Emond P., Gordon P., Pradat P.F., Praline J. (2010). ^1^H-NMR-based metabolomic profiling of CSF in early amyotrophic lateral sclerosis. PLoS One.

[10] Maillet S., Vion-Dury J., Confort-Gouny S., Nicoli F., Lutz N.W., Viout P., Cozzone P.J. (1998). Experimental protocol for clinical analysis of cerebrospinal fluid by high resolution proton magnetic resonance spectroscopy. Brain Res. Protoc..

[11] Fonteh A.N., Harrington R.J., Tsai A., Liao P., Harrington M.G. (2007). Free amino acid and dipeptide changes in the body fluids from Alzheimer's disease subjects. Amino Acids.

[12] Czech C., Berndt P., Busch K., Schmitz O., Wiemer J., Most V., Hampel H., Kastler J., Senn H. (2012). Metabolite profiling of Alzheimer’s disease cerebrospinal fluid. PLoS One.

[13] Mochizuki Y., Oishi M., Hara M., Takasu T. (1996). Amino acid concentration in dementia of the Alzheimer type and multi-infarct dementia. Ann. Clin. Lab. Sci..

[14] D’Aniello A., Fisher G., Migliaccio N., Cammisa G., D’Aniello E., Spinelli P. (2005). Amino acids and transaminases activity in ventricular CSF and in brain of normal and Alzheimer patients. Neurosci. Lett..

[15] Nicoli F., Vion-Dury J., Confort-Gouny S., Maillet S., Gastaut J.L., Cozzone P.J. (1996). Cerebrospinal fluid metabolic profiles in multiple sclerosis and degenerative dementias obtained by high resolution proton magnetic resonance spectroscopy. C. R. Acad. Sci. Paris.

[16] Stoop M.P., Coulier L., Rosenling T., Shi S., Smolinska A.M., Buydens L., Ampt K., Stingl C., Dane A., Muilwijk B. (2010). Quantitative proteomics and metabolomics analysis of normal human cerebrospinal fluid samples. Mol. Cell. Proteomics.

[17] Wishart D.S., Lewis M.J., Morrissey J.A., Flegel M.D., Jeroncic K., Xiong Y., Cheng D., Eisner R., Gautam B., Tzur D. (2008). The human cerebrospinal fluid metabolome. J. Chromatogr. B Anal. Technol. Biomed. Life Sci..

[18] Mandal R., Guo A.C., Chaudhary K.K., Liu P., Yallou F.S., Dong E., Aziat F., Wishart D.S. (2012). Multi-platform characterization of the human cerebrospinal fluid metabolome: a comprehensive and quantitative update. Genome Med..

[19] Ibáñez C., Simó C., Barupal D.K., Fiehn O., Kivipelto M., Cedazo-Mínguez A., Cifuentes A. (2013). A new metabolomic workflow for early detection of Alzheimer's disease. J. Chromatogr. A..

